# Two-dimensional Peripheral Refraction and Retinal Image Quality in Emmetropic Children

**DOI:** 10.1038/s41598-019-52533-7

**Published:** 2019-11-07

**Authors:** Weizhong Lan, Zhenghua Lin, Zhikuang Yang, Pablo Artal

**Affiliations:** 1 Aier School of Ophthalmology, Central South University, 410000 Changsha, China; 2 Aier School of Optometry and Vision Science, Hubei University of Science and Technology, Xianning, China; 3 Laboratorio de Óptica, Universidad de Murcia, Campus de Espinardo, 30100 Murcia, Spain

**Keywords:** Translational research, Applied optics

## Abstract

The relationship between the optical properties of the eye in the periphery and myopia development is still under debate. To further clarify this issue, we provide here baseline data of two-dimensional peripheral optics results in a group of emmetropic Chinese children. Peripheral aberrations were measured under cycloplegia by using an open-view Hartmann-Shack wavefront sensor (VPR, Voptica SL, Spain). This instrument allows to measure fast in the horizontal visual field from temporal 30° to nasal 30° every 1°. Two-dimensional (2D) maps were retrieved from a series of horizonal scans taken every 4° from 20° superior to 16° inferior covering a visual field of 60 × 36°. A relatively homogeneous pattern of the 2D relative peripheral refraction was found across all these emmetropic subjects. Using cluster analysis followed by manual visual refinement, the 2D maps were identified to fit into four categories. More than 70% of the subjects showed a nearly flat horizontal refraction with a slightly myopic shift in the superior retina. Peripheral astigmatism was quite constant across subjects and similar to that expected theoretically. Peripheral aberrations were also similar to those in the fovea for a large retinal area. These baseline data would offer an important reference to compare with the future evolution with time, as well as with other refractive or age groups of subjects, to better understand the role of peripheral optical properties in myopia development.

## Introduction

Myopia is a highly prevalent disease affecting millions of people worldwide^1^. In the last decades the number of myopic people increased largely in several parts of the world, in particular in east-Asia. The impact of myopia on public health is enormous and maybe even underestimated, because often only the costs of optical corrections are considered. Nevertheless, myopia is related to several blind-threating visual impairment, such as glaucoma, cataract, retinal degeneration and retinal detachment^2^. Thus, the need of a better understanding of the underlying causes of myopia that could ultimately lead to its prevention and control is very important.

A particular aspect in relation to the optics in myopic subjects that has attracted the attention of researchers for several decades is its peripheral behavior. The earliest observation of a large variation in the relative peripheral refraction (RPR) in humans was reported by Ferree *et al*.^3^. Since then, many studies have been performed on different aspects of the eye’s peripheral image quality^4–8^. This included the effect of accommodation^9^ and how the correction of aberrations affected visual performance in the periphery^10^. In the horizontal meridian, most studies showed a significant tendency to a relative hyperopia in the periphery of the myopic eyes. In addition, off-axis astigmatism was found to be the main aberration in the periphery for both myopes and hyperopes.

Measuring peripheral optics with enough accuracy poses some special challenges. This has been approached by the development of new custom-designed wavefront sensors allowing fast data acquisition^11^. This type of instruments has already been used to collect high resolution peripheral optics data in groups of healthy adults^12^ and patients after cataracts^13^. Despite of these instruments, there are still a lack of understanding with regard to the actual role of peripheral optical properties in myopia development. Firstly, there are not yet complete optical data, including not only refraction, but also full retinal image quality metrics. And probably more importantly, most of the previous works were conducted based on cross-sectional studies, which was unable to give a clear answer whether a particular peripheral optics is a cause or consequence of the myopization process. On the one hand, studies indicated that peripheral optics is more likely a consequence. For instance, due to a compensatory effect between defocus and oblique astigmatism, a similar amount of blur appears in the periphery, independently of the central refractive state. In addition, peripheral hyperopia in the horizontal meridian was reported to be unable to predict myopia progression^14^. These results may question the hypothesis that modifying the peripheral defocus alone could prevent the development of myopia. But on the other hand, there are some optical interventions showing a moderate clinical success to control myopia that claim to be based on the changes introduced on the peripheral optics.

To fully elucidate this important question, we determined to measure carefully and comprehensively the peripheral optical properties in centrally emmetropic children and follow up the change of these properties with time through a large longitudinal study. In this report, we present the high-resolution, wide-field two-dimensional data of peripheral refraction and optical aberrations obtained at the first stage of the study, expecting to serve as a baseline to compare with their evolution with time and with those from other groups of subjects with different central refractive status.

## Results

### Averaged 2D maps of peripheral retinal image quality

**Figure 1:**
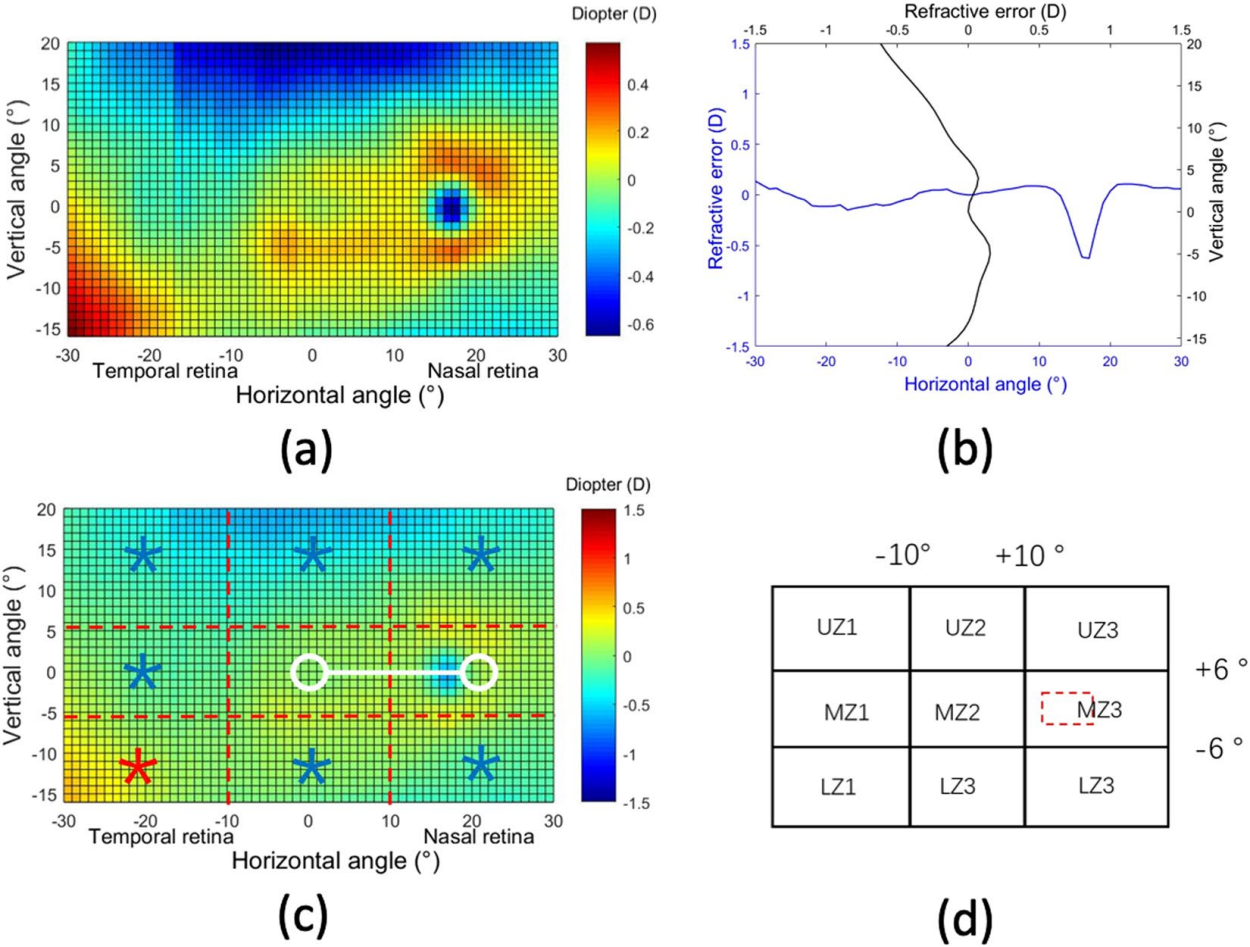
(**a**) Average 2D maps of the relative peripheral refraction. Positive and negative values in x-axis indicate nasal retinal and temporal retinal respectively. For y-axis, positive values represent superior retina and negative ones represent inferior retina. The color-code is in diopters. (**b**) Horizontal and vertical section of the average peripheral refraction depicted in panel (a). (**c**) Statistical analysis of the changes of RPR in eight zones with respect to the center. A zone with a white circle represents no statistical difference with the center, with blue star a statistical myopic difference and with a red star a statistically significant hyperopic difference. (**d**) Codes used for the different zones in the statistical analysis. The area of each box is 20 × 12 degrees. The red dotted-line represents the optics disc area not included in the analysis.

Figure 1a shows the color-coded two-dimensional (2D) maps of the relative peripheral refraction (RPR). This figure represents the averaged values from the complete cohort of subjects. In this group, the average RPR has a full range of less than 1 D in the area of 60 × 36°. Although the RPR tends to be flat, there is an area with a significant relative myopia in the superior retina (bluish region). It is interesting to note that our high-resolution measurements were even able to capture the features related to the optic nerve head (located around 17° in X-axis with a radius of 3°) indicating a relative myopic defocus of around + 0.75D. Figure 1b shows the horizontal and vertical central sections of the 2D map of Fig. 1a to better distinguish the actual defocus values. We have performed a statistical analysis of the RPR within the areas in the retina compared with the center (see methods section and supplementary material for additional details). The name codes for these areas is shown in Fig. 1(d). We did not found differences in refractive error between zones MZ2 (0.04 ± 0.28D) and MZ3 (0.09 ± 0.36D) (t = 1.4827, p = 0.142). Compared with the central MZ2 zone, most of the peripheral retina has a significant relative myopic defocus, except LZ1 having a statistically significantly hyperopic defocus. All values of the statistical analysis of the refractive error for each zone is presented as supplementary information.

**Figure 2:**
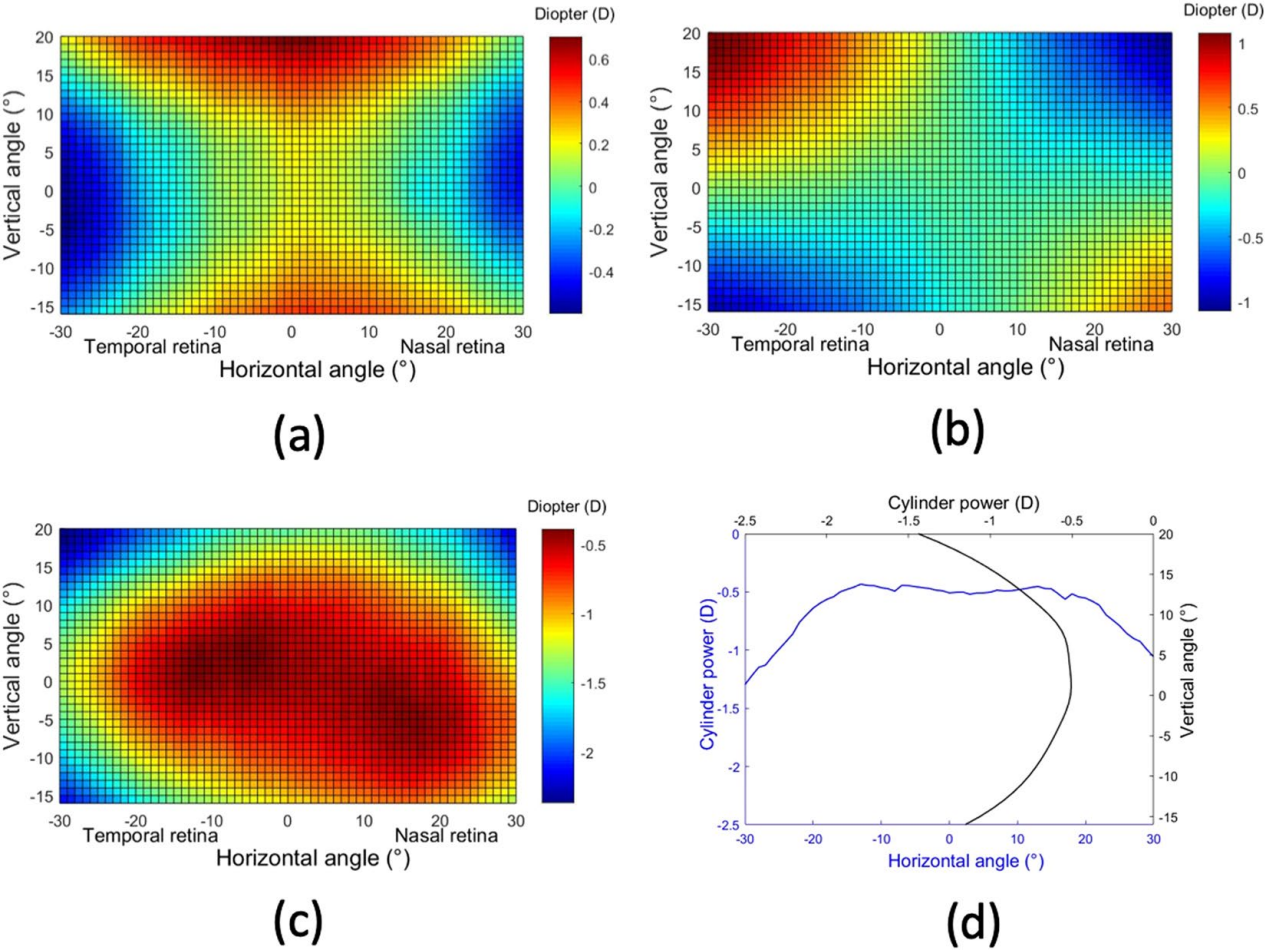
Average 2D maps of astigmatism. (**a**) J0 component. (**b**) J45 component. (**c**) Total astigmatism in a 2D map and corresponding horizontal and vertical sections (**d**). Positive and negative values in x-axis indicate nasal retinal and temporal retinal respectively. For y-axis, positive values represent superior retina and negative ones represent inferior retina. The color-code is in diopters.

Figure 2 shows the averaged values of astigmatism for each retinal eccentricity. The J0 component (Fig. 2a) changed mainly in the horizontal and vertical meridians. The value in the horizontal direction has the tendency to be more negative with eccentricity. In the vertical direction shows more positive value with eccentricity. The opposite distribution in these two directions caused a X-shape with a value near to zero in the central part. The J45 component of astigmatism (Fig. 2b) was like the 45° rotation counter-clockwise of J0. Figure 2c,d shows the total magnitude of astigmatism in a 2D map and the corresponding horizontal and vertical sections. These average results of astigmatism show the expected theoretical shape for oblique astigmatism^15^.

**Figure 3:**
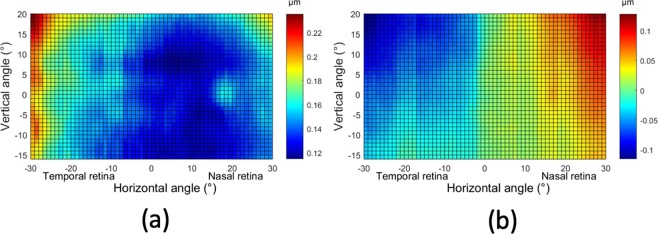
Average 2D maps of RMS of the high order aberrations (**a**) and coma (**b**). Positive and negative values in x-axis indicate nasal retinal and temporal retinal respectively. For y-axis, positive values represent superior retina and negative ones represent inferior retina. The color-code is in microns.

Figure 3a shows the 2D map of the root-mean squared (RMS) of the higher order aberrations, with the scale bar representing in this case microns. In this group, the amount of higher order aberrations is quite homogeneous in most of the central-nasal area (ranging from −7° to 25°). The values in the temporal retina appear to be slightly larger than the nasal retina, probably due to the common location of the kappa angle. In the vertical direction, values were similar for each meridian. As an example, Fig. 3b shows the 2D map corresponding to one single aberration: coma. It presents an interesting distribution similar to an isosceles triangle that has the bottom edge in the base of the map with vertex in the top-center. Coma has the tendency to increase from temporal side to nasal side. In the vertical direction, the value was decreasing from the inferior to the superior retina in temporal side, but this shift would conversely change in the nasal side.

### Classes of RPR maps

**Figure 4:**
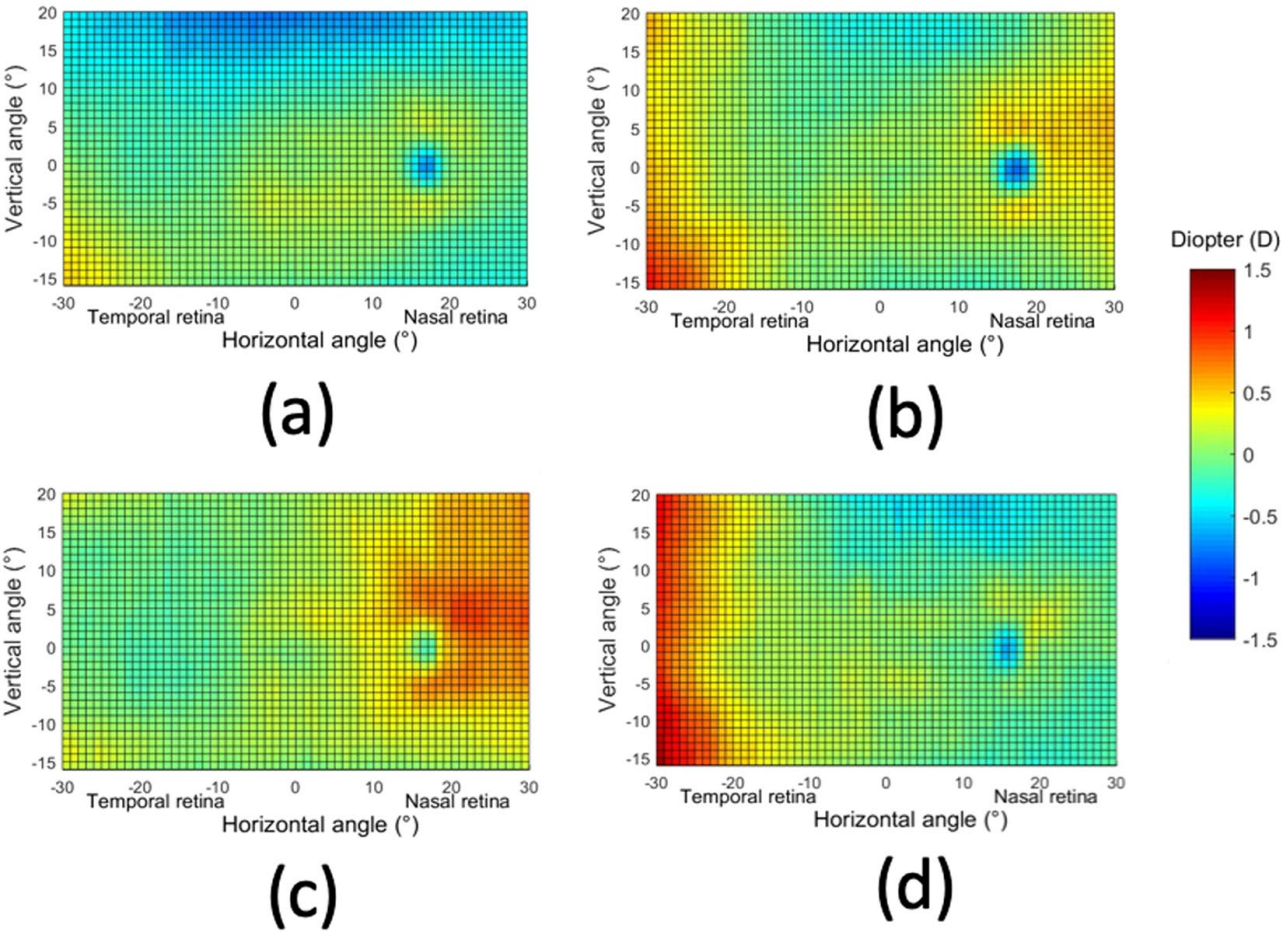
2D maps of the four classes of relative peripheral refraction maps. Category 1 (**a**) is the most commonly found (72%), category 2 (**b**), category 3 (**c**) and category 4 (**d**) account for the rest 14, 9 and 5% respectively. Positive and negative values in x-axis indicate nasal retinal and temporal retinal respectively. For y-axis, positive values represent superior retina and negative ones represent inferior retina. The color-code is in diopters.

**Figure 5:**
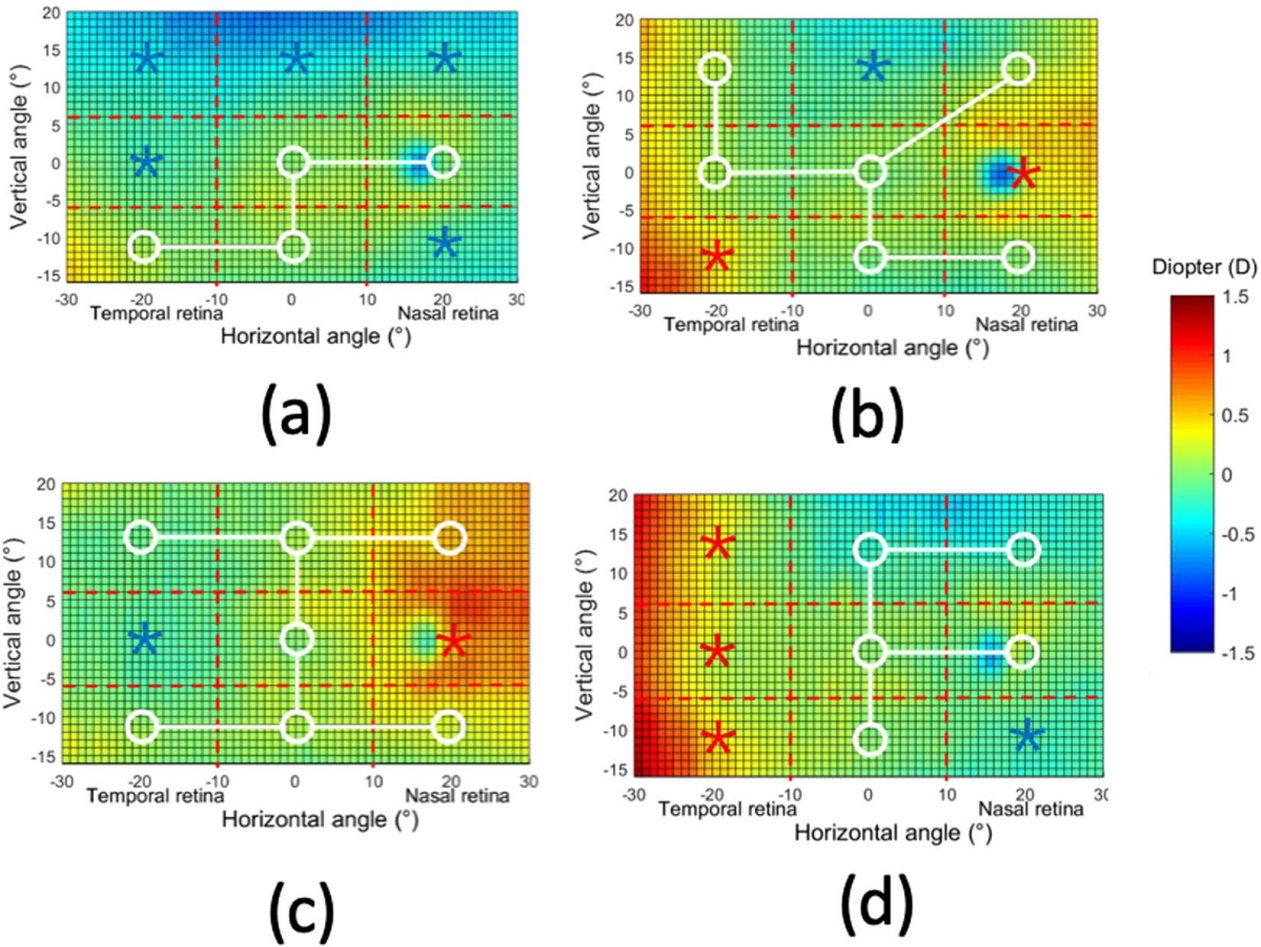
Statistical analysis of the changes of RPR in eight zones with respect to the center. A zone with a white circle represents no statistical difference with the center, with blue star a statistical hyperopic difference and with a red star a statistically significant myopic difference. The analysis is performed in the four identified categories of relative peripheral refraction maps. Category 1 (**a**), category 2 (**b**), category 3 (**c**) and category 4 (**d**). The color-code is in diopters.

By using the results of the relative peripheral defocus, we have performed an additional analysis based in finding the most common patterns in the 2D maps (see methods section for details of the cluster analysis performed). This allow us to determine the relative occurrence of the patterns in our group of subjects. We have identified four patterns that are represented in Fig. 4 (panels a–c). The most common pattern (category 1; C1) is that presented in panel (a). It occurred in the 72% of the subjects and has an averaged refraction in the whole area nearly emmetropic: 0.02 ± 0.27D. This map is similar to the average of Fig. 2 (although the scales in both figures are different). Figure 4b shows the second most common type of pattern (category 2; C2) that is present in 14%. The mean of refractive error in the whole area is −0.05 ± 0.32D. It presents hyperopic shifts in both sides of horizontal direction of the map (beyond 21° in nasal retina or outside 19° in temporal retina). Figure 4c shows the third type of identified pattern (category 3; C3) occurring in the 9% of participants with a mean refractive error of −0.06 ± 0.27D. A relative hyperopic defocus appeared in the nasal side from inferior −6° to the whole superior retina (Mean = 0.55D, STD = 0.43D). Finally, Fig. 4d shows the least common type of pattern (category 4; C4), only occurring in 5% of the subjects. The mean refractive error was 0.08 ± 0.45D and shows a distinct hyperopic area in the temporal visual field ranging from 21° to 30°. There were no significant difference in refractive error among the four categories. (ANOVA test, F = 0.261, p = 0.853). A full statistical analysis of the differences of RPR for the different retinal areas and the four categories was also performed. Figure 5 shows the results and all the statistical data for each category is presented as supplementary information.

**Figure 6:**
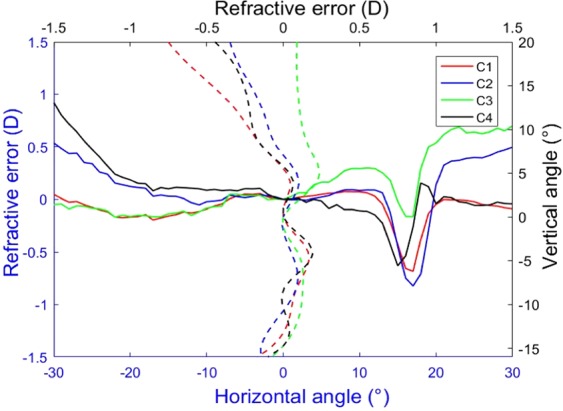
Horizontal and vertical sections of the 2D maps of the four categories depicted in Fig. 4. Positive and negative values in x-axis indicate nasal and temporal retina and in the y-axis (right) superior and inferior retina respectively.

To better compare the different maps for each category, Fig. 6 shows both the horizontal and vertical sections for each of the four categories. In the central horizontal meridian, the pattern of C1 is nearly flat (ignoring the area of the optics nerve head). In categories C2 and C4, there was a small hyperopic shift in the temporal side. In the central vertical meridian, the refraction profiles were quite similar for all the categories in the inferior part.

## Discussion

We have collected a large series of optical data in the periphery in a group of emmetropic children under controlled experimental conditions. We have used a fast and accurate scanning wavefront sensor to measure the optical aberrations in the horizontal meridians across 60 degrees of visual field. By controlling the vertical fixation, we were able to reconstruct 2D maps of different optical parameters covering an area of 60 × 36 degrees across the retina. From the wavefront aberrations, we retrieved for each retinal location in high resolution refraction (defocus and astigmatism) and higher order aberrations. The group of subjects was very homogeneous, both in age, central refractive state and ethnicity. This has a significant advantage to determine the natural optical properties in the periphery before they are affected by myopia progression.

Previous studies on the peripheral optics presented a significant variability among different subjects^16^. This fact could be interpreted as having many initial shapes for each individual eye or a scenario starting from a more or less common shape that could be modified during refractive development. Our initial hypothesis was that most children in a homogeneous group with similar emmetropic refractive errors would also have similar optical properties in the periphery.

In order to avoid confounding factors, we have also collected all data with paralyzed accommodation by performing the measurements under cycloplegia.

A new, and important feature, of this work is the capability of obtaining a complete map of the two-dimensional, retina in a relatively large field of view comprising 60 × 36 degrees around the fovea. This complete most of the previously available results that were typically restricted to the horizontal meridian only.

We found that most subjects presented very similarly flat maps of peripheral refraction when compared with the central refraction. In most subjects, the refraction in the horizonal meridian was exactly as in the fovea, while in the superior vertical meridian there was a hyperopic shift reaching an average value of 0.75D at 20 degrees. In a fraction of the subjects smaller than 20% there was some myopic shift smaller than 1 D at 30 degrees.

We have identified the most commonly occurring patterns of relative peripheral refraction. Over 70% of subjects presented the flat pattern with slightly myopic superior retina. For the rest of the subjects, there were smaller deviation towards hyperopic (around 0.5 D at 30 degrees) in the horizontal meridian. All subjects have a plano-refraction in the inferior part of the retina.

As expected for subjects without central astigmatism, the component of peripheral astigmatism J0 changed mainly along with the horizontal (axis 0°) and vertical meridians (axis 90°) while J45 changed along with the meridians of 45° and 135°. Concerning the higher order aberrations, there were very similar to those of the fovea in a large area of the retina. It should be noted that these subjects presented a small amount of higher order aberrations (less than 0.2 microns for a 4-mm pupil diameter in an area of 40 × 30 degrees).

**Figure 7:**
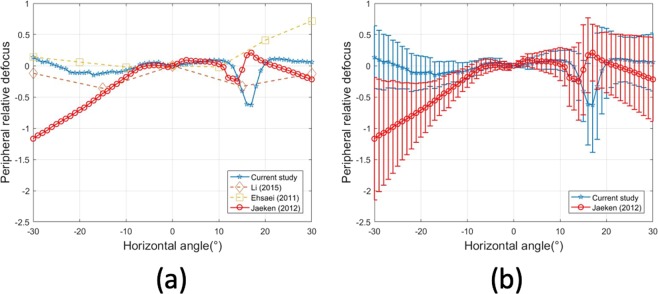
(**a**) Comparison of relative peripheral refraction results from several studies. (**b**) Results for Jaeken & Artal (2012) and this work including error bars representing the standard deviation of the measurements.

It is interesting to compare our results with others previously published. Since there are not full retina data, we have restricted the comparison to the horizontal section in Fig. 7. Figure 7a shows the average results. The current study reported a rather flat refraction in the periphery (except for the area of the optic disc). A study in a large population in Chinese students^17^ measured only a few eccentricities with a clinical autorefractometer reported also small values at these eccentricities, perhaps due to an averaging effect. Another study in a smaller group also with an autorefractometer including Caucasian and Asian adults also demonstrated a similar pattern^18^. By using a version of the instrument used in this study, Jaeken & Artal^12^ measured a group of emmetropic adults showing an average myopic shift in the temporal side.

Perhaps, even more interesting is to compare the variability among subjects in the groups. We could do this comparison directly only with the study of ref.^12^. Figure 7b shows the standard deviation in the relative peripheral refraction as error bars. The variability on the group of adults is much larger than what was found in our study with children.

As far as we know, there are only one previous study reporting 2D peripheral refraction results in a small group of emmetropic adults^19^, but they used a commercial wavefront sensor with a much smaller sampling resolution given by the selection of fixation points.

Although we found a homogeneous relative peripheral refraction in this populations, the subtle differences may indicate some tendency for myopia development. This should be clarified with the future follow-up of these subjects. These results may suggest a quite common initial condition for the peripheral optics near spherical emmetropia in a large area of the retina, but with a significant astigmatism. From this optimized optical situation, eyes may evolve to different situations depending the particular refractive changes.

In conclusion, we measured peripheral optics quality with high resolution within a large retinal area of more than 2160 squared degrees in a group of emmetropic children. Contrary to some previous ideas, we reported that the optics in the periphery was very similar in most of the emmetropic children analyzed. We found that most children has similar patterns of the 2D relative peripheral refraction. The most commonly occurring case in more than 70% of subjects was a nearly flat horizontal refraction with a slightly myopic shift in the superior retina. Peripheral astigmatism was also quite constant across subjects and similar to that expected theoretically. Peripheral aberrations were also similar to that in the fovea for a large retinal area. The results obtained in this work may serve as a baseline for future studies to better understand the role of peripheral optics in myopia progression and to develop possible optical control strategies.

## Methods

### Subjects

A group of 82 emmetropic (0.03 ± 0.28D, male 48.8%) Chinese children without any known ocular disease and with age ranging from 10 to 14 years (12.32 ± 1.12) were enrolled in the study. Detailed demographic and optical information on the population is presented in Table 1. The participants were students in one single school in a rural area of the Hunan province in China. All participants, and their parents, were informed and signed the consent before the trial was started and all examinations followed the tenets of declaration of Helsinki. Registry and data collection procedures have been approved by the Committee of Research Ethics of the Aier School of Ophthalmology, Central South University.

**Table 1:** General information on the subjects.

	Age (years)	SER	C	J0	J45	AL (mm)	K1	K2
Total (n = 82) Mean (SD) [95% CI]	12.32 (1.12) [10.12, 14.51]	−0.03 (0.28) [−0.57, 0.52]	−0.51 (0.23) [−0.97, −0.05]	0.18 (0.16) [−0.14, 0.5]	−0.1(0.1) [−0.3, 0.09]	23.15 (0.7) [21.77, 24.53]	42.77 (1.54) [39.74, 45.8]	43.52 (1.62) [40.34, 46.7]
Male (n = 40) Mean (SD) [95% CI]	12.53(1.24) [10.09, 14.96]	0(0.28) [−0.54, 0.55]	−0.56(0.24) [−1.02, −0.1]	0.21(0.16) [−0.1, 0.53]	−0.1(0.12) [−0.31, 0.11]	23.33(0.71) [21.93, 24.72]	42.45(1.43) [39.65, 45.26]	43.23 (1.54) [40.21, 46.25]
Female (n = 42) Mean (SD) [95% CI]	12.12 (0.97) [10.22, 14.01]	−0.05 (0.28 [−0.6, 0.49]	−0.46 (0.23) [−0.91, −0.02]	0.15 (0.16) [−0.17, 0.46]	−0.1 (0.09) [−0.29, 0.08]	22.98 (0.66) [21.68, 24.28]	43.07 (1.64) [39.85, 46.29]	43.8 (1.7) [40.45, 47.15]

SER: Spherical equivalent; C: Cylinder power; AL: axial length

J0 and J45: power of the two Jackson cross-cylinder component

K1: flat cornea curvature; K2: step cornea curvature.

### Instrument & measurement procedure

Peripheral refraction and aberrations were measured by using an open-view Hartmann-Shack wavefront sensor (Voptica Peripheral Refractor, VPR, Voptica SL, Murcia, Spain). The details of the instrument had been published elsewhere^11^. The device scans instead of having the subject moving his eye to fixate at different angles. The path length between the eye and the first element of the sensor is constant at each measuring angle. There are no moving elements in front of the subject’s eye. The illumination light is in the near infrared and custom software was developed to control and synchronize the sensor movements with image acquisition and to perform data processing. The system is a combination of a moving part and a static part. The moving part uses a rotation stage, a caging system and a high speed camera. The near infrared 780 nm measuring light is coupled onto the moving arm by means of an optical fiber. The fixed part of the instrument contains a custom made large mirror and a hot mirror in front of the subject’s eye allowing the instrument to operate in open field. This instrument allows to measure the wavefront aberration of the central 60° of horizontal visual field every 1° in 1.3 seconds for each scan including 61 Hartmann-Shack images. The frequency of the camera is 60 fps while the motor and the controller allow a maximum speed of 80°/second. The camera and motor were synchronized, so the frequency on the camera is 50 fps and the speed of the motor is 50°/second. This operating time is short enough to avoid the effects of tear film break-up during the measurements^20^. The inter-subject variability during the measurements showed an averaged standard deviation for the whole measured visual field of 0.13D.

**Figure 8:**
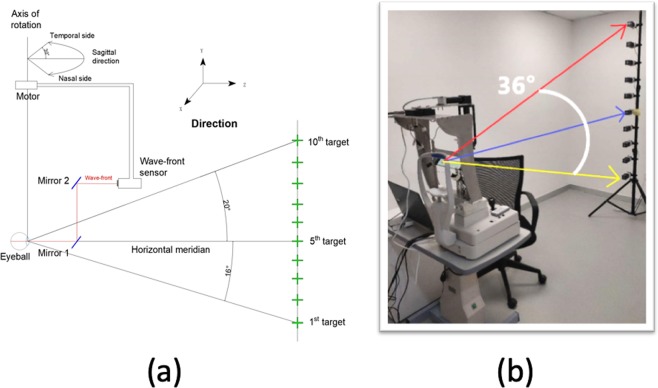
(**a**) Schematic diagram of the set-up used to measure peripheral optics. (**b**) Actual picture of the instrument and experimental arrangement for vertical fixation.

In order to obtain also measurements in the vertical and oblique sections, we asked the subjects to fixate to a series of 10 targets arranged vertically at 2.5 meters. Figure 8a shows an schematic diagram of the experimental setup. The vertical range of measurement is limited to 20° (up to the 10th target) in the superior side of retina to 16° (the 1st target) in the inferior side of retina within intervals of 4°. Subjects under cycloplegia were asked to fixate from the 1st cross to the 10th cross in sequence in a dark room. For each vertical fixation, series of horizonal scans were recorded. Every Hartmann-Shack image was analyzed within a diameter of 4 mm to estimate the aberrations expressed as a Zernike polynomials expansion. Zernike coefficients are fitted over a circle encircling all measured spots before they are rescaled mathematically to a pupil size smaller than the minor axis of an ellipse fitted around the measured spots. For each retinal location, the mean of four measurements was obtained and the refraction is calculated from the second order terms. Only the right eye of every subject was measured to avoid confounding factors. Meanwhile, the left eye was covered during the trial. Figure 8b shows a real picture of the experimental setup with an inset presenting one of the participants during the measurements session. Mydriasis was induced by one drop of Alcaine (Alcon, Japan), followed by 2 drops of 1% Cyclopentolate (Alcon, Japan) administered 5 minutes apart, for both eyes. Pupillary light reflex was checked after 30 minus of the last administration of cyclopentolate. If the light reflex remained, one additional drop of cyclopentolate was applied and the following measurement was not conducted until mydriasis was obtained. Axial length and cornea curvature were measured with LENSTAR LS 900 (Haag-Streit AG, Koeniz, Switzerland).

### Data analysis

Two-dimensional (2D) maps were obtained from the results of 10 horizontal sections (610 points in the retina) by using customized scripts in MATLAB (MathWorks, Natick, USA). For all 2D maps, positive value indicates nasal side and negative value indicates temporal side in abscissas. In the ordinates, a positive value indicates the location of superior retina and a negative value indicates the location of inferior retina. Relative peripheral refraction (RPR) maps were generated by subtracting the central refraction (SE) at the fovea. Other 2D maps for different parameters, such as astigmatism, or higher order aberrations, were produced accordingly.

We have also performed a classification of 2D RPR maps. This was first carried out by using a statistical cluster analysis method with a final visual evaluation. The procedure of classification contained 2 steps. First, a cluster analysis was applied to preset objective categories as reference to initiate the classification. The function of weighted average distance (WPGMA) was applied for the cluster analysis. Before running the function, the value was normalized as centered, with mean as 0 and the standard deviation as 1 for the corresponding position of retina of all participants by using the followed formulation (X-MEAN(X))/STD(X). The distance between each pair of observations (SER value in the whole 2D map) was computed using Euclidean distance. Based on the distances, a dendrogram of cluster analysis was produced and 6 categories were established. Then, 3 researchers manually refined the classification by visualizing the 2D maps of peripheral relative refraction. During the refinement process, 2 of the 6 categories were merged into other categories because of the homogeneity with other groups. In addition, the data of 1 child were found as an outlier and excluded, due to the difficulty of the determination of category assigned. Thus, 4 categories for 81 subjects were determined.

Within each 2D RPR map, a statistical analysis was performed to determine the differences in refraction between the peripheral zones and the center. Each 2D map was divided into multiple zones. Horizontally, the map was evenly divided into 3 areas. Since the vertical measurement range was from superior 20° to inferior 16°, the 2 interval points was set superior 5.5° and inferior 5.5°, to achieve a compromise between the relative even area size vertically and the middle part centering on the fovea. Therefore, the map was divided into 3 × 3 zones. The average refractive error was calculated for each specific zone. In addition, the data of the optic nerve head near area (horizon: nasal 12.5° to 20.5°, vertical: superior 4° to inferior 4°) were excluded for the analysis. See supplementary material for a graphical description of this partition. Descriptive data of each regions was obtained by running customized code based on Matlab R2016 a (MathWorks, US). Paired-t test was used to evaluate the refractive difference between peripheral zones and central zone (Matlab). Power of test was calculated by using PASS 11 (NCSS, US). One way ANOVA analysis was used to compare the refractive error among categories by using SPSS 20.0.

## Supplementary information

Statistical analysis of the peripheral differences

## Data Availability

The datasets and codes used within this paper are available from the corresponding author upon reasonable request.
