# Supplementary material for: Two-dimensional Peripheral Refraction and Retinal Image Quality in Emmetropic Children
Source: Sci Rep. 2019 Nov 7;9:16203. doi: 10.1038/s41598-019-52533-7 (PMC6838170; doi:10.1038/s41598-019-52533-7)
Supplement: Supplementary file 1 — Statistical analysis of the peripheral differences [file 41598_2019_52533_MOESM1_ESM.doc]

***Two-dimensional Peripheral Refraction and Retinal Image Quality in Emmetropic Children***

**Weizhong Lan^1,2^, Zhenghua Lin^1^, Zhikuang Yang^1,2*^ and Pablo Artal^1,3^***

Aier School of Ophthalmology, Central South University, 410000 Changsha, China^1^

Aier School of Optometry and Vision Science, Hubei University of Science and Technology, Xianning, China^2^

Laboratorio de Óptica, Universidad de Murcia, Campus de Espinardo, 30100 Murcia, Spain^3^

* Corresponding authors: Yangzhikuan@aierchina.com & pablo@um.es

**Supplementary Information**

***Statistical analysis of the peripheral differences in refraction: procedure and data***

We performed a statistical analysis of the local changes in refraction with respect to the center in the 2D maps. The 2D RPR maps were divided into multiple zones. Horizontally, the map was evenly divided into 3 areas. Since the vertical measurement range was from superior 20° to inferior 16°, the 2 interval points was set superior 6° and inferior 6°, to achieve a comprise between the relative even area size vertically and the middle part centering on the fovea. Therefore, the map was divided into 3X3 zones. The average refractive error was calculated for each specific zone. In addition, the data of the optic nerve head near area (horizontal: nasal 12.5° to 20.5°, vertical: superior 4° to inferior 4°) were excluded for the analysis. Figure 1d in manuscript shows a schematic representation of the partition of the maps.

Table S1 presents the statistical results of the SER (spherical equivalent) for each zone for the average map (Fig 1d in manuscript). Tables S2 to S5 presents the data for the four categories presented in figure 5 of the manuscript.

| Table S1. All subjects (n=82) | | | | | | | | |
| --- | --- | --- | --- | --- | --- | --- | --- | --- |
| SER | Mean | SD | Median | 95%CI | | Power | t | p |
|  |  |  |  | Lower Limit | Upper Limit |  |  |  |
| MZ2 | 0.0379 | 0.2787 | 0.0285 | -0.51 | 0.58 | - | - | - |
| UZ1 | -0.2788 | 0.4559 | -0.2750 | -1.1723 | 0.6147 | 100% | -7.8826 | 0.0000 |
| UZ2 | -0.3326 | 0.3942 | -0.3547 | -1.1052 | 0.4401 | 100% | -13.7501 | 0.0000 |
| UZ3 | -0.1448 | 0.4478 | -0.1276 | -1.0224 | 0.7328 | 99.31% | -4.4208 | 0.0000 |
| MZ1 | -0.0664 | 0.3481 | -0.0332 | -0.7486 | 0.6158 | 94.68% | -3.5734 | 0.0000 |
| MZ3 | 0.0863 | 0.3582 | 0.05 | -0.6158 | 0.7884 | 31.65% | 1.4827 | 0.1420 |
| LZ1 | 0.1450 | 0.3906 | 0.2104 | -0.6206 | 0.9107 | 71.73% | 2.5362 | 0.0131 |
| LZ2 | -0.0056 | 0.2770 | 0.0028 | -0.5486 | 0.5373 | 57.68% | -2.1536 | 0.0342 |
| LZ3 | -0.1235 | 0.3462 | -0.1348 | -0.8020 | 0.5550 | 99.62% | -4.6306 | 0.0000 |

| Table S2. C1 (n=59) | | | | | | | | |
| --- | --- | --- | --- | --- | --- | --- | --- | --- |
| SER | Mean | SD | Median | 95%CI | | Power | t | p |
|  |  |  |  | Lower Limit | Upper Limit |  |  |  |
| MZ2 | 0.0330 | 0.2723 | 0.0249 | -0.5006 | 0.5666 | - | - | - |
| UZ1 | -0.4038 | 0.3773 | -0.3591 | -1.1434 | 0.3358 | 100% | -11.4979 | 0.0000 |
| UZ2 | -0.4093 | 0.3729 | -0.3780 | -1.1402 | 0.3215 | 100% | -16.7758 | 0.0000 |
| UZ3 | -0.2697 | 0.3606 | -0.2463 | -0.9764 | 0.4370 | 100% | -8.9949 | 0.0000 |
| MZ1 | -0.1176 | 0.3028 | -0.0777 | -0.7111 | 0.4759 | 99.74% | -4.7583 | 0.0000 |
| MZ3 | -0.0120 | 0.2774 | -0.0103 | -0.5556 | 0.5317 | 35.83% | -1.5961 | 0.1159 |
| LZ1 | 0.0995 | 0.3589 | 0.1847 | -0.6040 | 0.8030 | 77.32% | 1.3588 | 0.1795 |
| LZ2 | -0.0104 | 0.2704 | 0.0001 | -0.5403 | 0.5194 | 40.56% | -1.7240 | 0.0900 |
| LZ3 | -0.1936 | 0.3225 | -0.1851 | -0.8257 | 0.4385 | 100% | -5.5983 | 0.0000 |

| Table S3. C2 (n=11) | | | | | | | | |
| --- | --- | --- | --- | --- | --- | --- | --- | --- |
| SER | Mean | SD | Median | 95%CI | | Power | t | p |
|  |  |  |  | Lower Limit | Upper Limit |  |  |  |
| MZ2 | 0.0114 | 0.3077 | 0.0125 | -0.5916 | 0.6144 | - | - | - |
| UZ1 | 0.0832 | 0.3883 | 0.1397 | -0.6780 | 0.8444 | 21.78% | 1.1773 | 0.2663 |
| UZ2 | -0.2047 | 0.3160 | -0.3252 | -0.8241 | 0.4148 | 99.79% | -4.8141 | 0.0001 |
| UZ3 | 0.1449 | 0.3443 | 0.1187 | -0.5298 | 0.8197 | 50.4% | 1.9709 | 0.0770 |
| MZ1 | 0.1321 | 0.3938 | 0.1956 | -0.6397 | 0.9040 | 56.82% | 2.1325 | 0.0588 |
| MZ3 | 0.3164 | 0. 3436 | 0.1827 | -0.3570 | 0.9898 | 100% | 6.0111 | 0.0000 |
| LZ1 | 0.3751 | 0.3549 | 0.3074 | -0.3204 | 1.0707 | 99.7% | 4.7015 | 0.0001 |
| LZ2 | -0.051 | 0.3167 | -0.1483 | -0.6719 | 0.5698 | 23.18% | -1.2245 | 0.2488 |
| LZ3 | 0.0204 | 0.3510 | -0.1328 | -0.6676 | 0.7083 | 5.15% | 0.1158 | 0.9101 |

| Table S4. C3 (n=7) | | | | | | | | |
| --- | --- | --- | --- | --- | --- | --- | --- | --- |
| SER | Mean | SD | Median | 95%CI | | Power | t | p |
|  |  |  |  | Lower Limit | Upper Limit |  |  |  |
| MZ2 | 0.0792 | 0.2750 | 0.1223 | -0.4598 | 0.6182 | - | - | - |
| UZ1 | -0.1049 | 0.4185 | -0.0212 | -0.9251 | 0.7153 | 44.1% | -1.8100 | 0.1203 |
| UZ2 | 0.0594 | 0.3732 | 0.0271 | -0.6721 | 0.7909 | 5.48% | -0.2039 | 0.8452 |
| UZ3 | 0.5155 | 0.4857 | 0.4051 | -0.4364 | 1.4675 | 55.87% | 2.1080 | 0.0796 |
| MZ1 | -0.1632 | 0.3490 | 0.0095 | -0.8471 | 0.5208 | 95.53% | -3.6579 | 0.0106 |
| MZ3 | 0.5992 | 0.4138 | 0.6277 | -0.2119 | 1.4103 | 87.65% | 3.1175 | 0.0206 |
| LZ1 | -0.0182 | 0.4523 | 0.0892 | -0.9048 | 0.8684 | 11.78% | -0.7572 | 0.4776 |
| LZ2 | 0.0339 | 0.3251 | 0.2319 | -0.6034 | 0.6711 | 10.98% | -0.7127 | 0.5028 |
| LZ3 | 0.2020 | 0.3414 | 0.1819 | -0.4672 | 0.8712 | 19.45% | 1.0945 | 0.3157 |

| Table S5. C4 (n=4) | | | | | | | | |
| --- | --- | --- | --- | --- | --- | --- | --- | --- |
| SER | Mean | SD | Median | 95% CI | | Power | t | *p* |
|  |  |  |  | Lower Limit | Upper Limit |  |  |  |
| MZ2 | 0.1415 | 0.4125 | 0.1195 | -0.6671 | 0.9501 | - | - | - |
| UZ1 | 0.4780 | 0.3784 | 0.5740 | -0.2637 | 1.2197 | 99.18% | 4.3597 | 0.0223 |
| UZ2 | -0.1117 | 0.4599 | 0.0368 | -1.0131 | 0.7897 | 50.54% | -1.9738 | 0.1429 |
| UZ3 | -0.1009 | 0.5210 | 0.0907 | -1.1222 | 0.9203 | 36.06% | -1.6029 | 0.2073 |
| MZ1 | 0.4352 | 0.3225 | 0.3880 | -0.1970 | 1.0673 | 88.19% | 3.1440 | 0.0515 |
| MZ3 | 0.0449 | 0.4827 | 0.0593 | -0.9012 | 0.9909 | 39.67% | -1.6988 | 0.1879 |
| LZ1 | 0.6376 | 0.1714 | 0.5606 | 0.3016 | 0.9736 | 87.84% | 3.1271 | 0.0522 |
| LZ2 | 0.1132 | 0.2868 | 0.0982 | -0.4489 | 0.6753 | 7.35% | -0.4507 | 0.6828 |
| LZ3 | -0.0891 | 0.3916 | -0.0180 | -0.8565 | 0.6784 | 98.35% | -4.0911 | 0.0264 |
